# Event-related EEG oscillatory responses elicited by dynamic facial expression

**DOI:** 10.1186/s12938-021-00882-8

**Published:** 2021-04-27

**Authors:** Tuba Aktürk, Tom A. de Graaf, Yasemin Abra, Sevilay Şahoğlu-Göktaş, Dilek Özkan, Aysun Kula, Bahar Güntekin

**Affiliations:** 1 Program of Electroneurophysiology, Vocational School, Istanbul Medipol University, Istanbul, Turkey; 2 Program of Neuroscience Ph.D, Graduate School of Health Sciences, Istanbul Medipol University, Istanbul, Turkey; 3 Department of Cognitive Neuroscience, Faculty of Psychology and Neuroscience, Maastricht University, Maastricht, Netherlands; 4 Department of Biological Sciences, Faculty of Arts and Sciences, Middle East Technical University, Ankara, Turkey; 5 Meram Faculty of Medicine, Konya Necmettin Erbakan University, Konya, Turkey; 6 Department of Molecular Biology and Genetics, Faculty of Science, Sivas Cumhuriyet University, Sivas, Turkey; 7 Department of Biophysics, School of Medicine, Istanbul Medipol University, Istanbul, Turkey; 8 Regenerative and Restorative Medicine Research Center (REMER), Istanbul Medipol University, Istanbul, Turkey; 9 Institute for Psychology, Faculty of Human Sciences, Universität Der Bundeswehr München, Munich, Germany; 10 Department of Psychology, Faculty of Psychology and Educational Sciences, Ludwig-Maximilians-Universität München, Munich, Germany

**Keywords:** Event-related oscillations, Dynamic facial expression, Event-related power analysis, Emotion

## Abstract

**Background:**

Recognition of facial expressions (FEs) plays a crucial role in social interactions. Most studies on FE recognition use static (image) stimuli, even though real-life FEs are dynamic. FE processing is complex and multifaceted, and its neural correlates remain unclear. Transitioning from static to dynamic FE stimuli might help disentangle the neural oscillatory mechanisms underlying face processing and recognition of emotion expression. To our knowledge, we here present the first time–frequency exploration of oscillatory brain mechanisms underlying the processing of dynamic FEs.

**Results:**

Videos of joyful, fearful, and neutral dynamic facial expressions were presented to 18 included healthy young adults. We analyzed event-related activity in electroencephalography (EEG) data, focusing on the delta, theta, and alpha-band oscillations. Since the videos involved a transition from neutral to emotional expressions (onset around 500 ms), we identified time windows that might correspond to face perception initially (time window 1; first TW), and emotion expression recognition subsequently (around 1000 ms; second TW). First TW showed increased power and phase-locking values for all frequency bands. In the first TW, power and phase-locking values were higher in the delta and theta bands for emotional FEs as compared to neutral FEs, thus potentially serving as a marker for emotion recognition in dynamic face processing.

**Conclusions:**

Our time–frequency exploration revealed consistent oscillatory responses to complex, dynamic, ecologically meaningful FE stimuli. We conclude that while dynamic FE processing involves complex network dynamics, dynamic FEs were successfully used to reveal temporally separate oscillation responses related to face processing and subsequently emotion expression recognition.

## Background

Recognition of facial expressions (FE) is central to human social interactions. Facial expressions of basic emotions can be recognized irrespective of culture or geographical location [1–3], underlining the evolutionary value of recognizing others' emotions and inferring their intentions. This ability to recognize emotions in FEs can be impaired in certain pathologies, including Alzheimer's disease [4–8], schizophrenia [9–11], or autism spectrum disorders [12–14], and is subject to change throughout the lifespan [15, 16]. Given its fundamental social function, it is important to understand what the neural mechanisms of FE processing are. Deeper understanding might provide opportunities to develop more comprehensive treatment approaches for patients with impairments in FE recognition.

Most empirical studies on the processing of facial expressions have used static images of FEs. These studies revealed that brain oscillations are sensitive to emotional processes (for reviews, see Knyazev [17] and Güntekin & Başar [18]). Unsurprisingly, the neural response to an image of an emotional face is complex and multifaceted, involving aspects of basic visual processing, face processing, and ultimately emotion processing. In the frequency domain, different frequency bands might reflect different components of FE processing, and these are widely distributed over the scalp aside from certain epicenters [18, 19]. Several studies [19–21] linked event-related delta activity to the perception of emotional pictures [18, 22]. Event-related delta and theta oscillations are stronger in response to emotional than neutral FEs [18, 20, 21, 23, 24] and seem to be involved in both non-conscious and conscious aspects of FE processing [20]. It has been shown that anterior theta and posterior delta are involved in FE processing [19, 21] with oscillatory results being subject to change topologically, depending on the particular FE paradigms used [19]. Dravida et al. [25] suggested that increased theta oscillations in response to FE stimuli may originate from the occipital face area. And several studies showed that alpha oscillations also play an essential role during FE processing [18, 26–29]. Balconi et al. [27, 29] reported that emotional stimuli with high valence and arousal value compared to neutral stimuli entailed reduced alpha power [27, 29]. In contrast, Güntekin and Başar [26] showed increased temporoparietal alpha oscillations in response to angry FE.

In sum, while there is extensive previous research linking a broad range of oscillatory mechanisms to the processing of FEs, the precise neural mechanisms underlying different components of face processing and emotion recognition remain unclear. Moreover, real-life FEs are dynamic [3], not static, and FE recognition is a process with temporal progression: it evolves over time. Dynamic stimuli are, inherently, more complex than simple images. Yet in this instance, an exploration of the oscillatory response over time, as participants view the development of an emotional facial expression over time, might help disentangle different components of the oscillatory response previously lumped together in studies with static FE stimuli. Here, we used time–frequency analysis of EEG responses to dynamic FE stimuli that started neutral, and either remained neutral (control condition) or developed a joyful or a fearful expression.

To our knowledge, no prior studies used EEG to study the oscillatory basis of dynamic FE processing. Two behavioral studies compared dynamic FEs to static FEs [30, 31], revealing stronger perception and emotional responses to dynamic FEs as compared to static FEs. And there were several studies investigating dynamic FEs with EEG, but focusing on event-related potentials [32–35]. Recio et al. [32–34] showed a differentiated response in early posterior negativity and the late positive complex during the processing of dynamic FEs in comparison with static FEs. In these studies, it has been shown that the difference between the response to emotional vs. neutral FEs is more evident in dynamics FE in both early and late ERP components. Here, we evaluated whether a temporally separated oscillation response might be found in frequency bands previously associated with static FE processing; delta, theta, and alpha band. As mentioned, a key aspect of dynamic FE processing is its temporal dynamics. By separating the onset of the face presentation, and the development of an emotion expression, we might differentiate different aspects of the static FE oscillation responses previously reported. Based on previous research, we expected that the more pronounced response in later time windows will correspond to the FE with the highest arousal level. To test these hypotheses, we recorded EEGs of 18 healthy young participants as they viewed dynamic facial expression video clips. FEs were presented in 3 different FE categories (fearful, joyful, and neutral). Event-related power, as well as phase-locking values, were analyzed over time for the delta (1–3, 5 Hz), theta (4–7 Hz), and alpha (8–13 Hz) bands. For statistical analyses, we zoomed in on two time windows displaying distinct responses, around 0 ms (first TW) and 1000 ms (second TW), putatively corresponding to neural mechanisms of face processing and emotion expression recognition, respectively.

## Results

### Valence, arousal, and FE recognition scores

We first evaluated behavioral and subjective responses to the dynamic FE stimuli. There were 12 videos in TOTAL, 4 from each category; neutral, joyful, or fearful. FE recognition scores were based on the correct classification of each of the videos into their category, and for any given category could, therefore, range from 0 to 4 (correctly classified videos out of the 4). Valence (mood upon seeing the dynamic FE stimuli) and arousal (excitation by the stimuli) scores ranged from 1 to 9, and were based on a subjective self-evaluation scale after seeing each video again after the main measurement had been completed (see Methods).

**Fig. 1:**
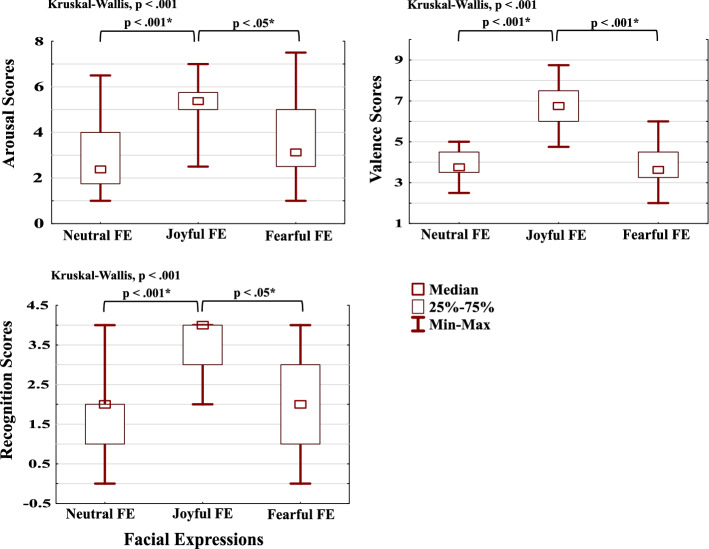
Box plots of the Kruskal–Wallis test for scores of the valence, arousal, and FE recognition. Asterisks (*) stands for significant pairwise comparisons. FE: facial expression, SE: standard error, SD: standard deviation. The maximum possible recognition score was four points for each FE. Valence scores and arousal scores ranged from 1 to 9 (negative to positive and low to high excitation, respectively, see Methods for details)

Valence scores, arousal scores, and FE recognition scores, all differed across the three categories of videos (neutral, joyful, fearful, *p* < 0.001, see Fig. 1). Pairwise comparisons showed that joyful FE had the highest valence, arousal, and recognition scores (*p* < 0.05, Bonferroni-adjusted, two-sided). These results confirm that dynamic FE stimuli from different categories affected participants differently and were recognized as conveying different emotions. It then becomes interesting to assess the potentially different neural responses to these categorical FEs, focusing in this study on the event-related oscillatory responses in delta, theta, and alpha frequency bands.

### Event-related oscillatory activity during dynamic FE processing

As discussed in the Introduction, most prior studies used static FE stimuli to study neural mechanisms underlying the processing of facial expressions. Since the neural correlates of dynamic FE processing are comparatively unexplored, we here concretely focused on three frequency bands, previously linked to (different aspects of) FE processing; delta, theta, and alpha. For each, we present time–frequency plots for (1) power analysis which shows the time-locked and/or phase-locked responses to the presentation of dynamic FE videos and (2) phase-locking values which quantify the phase angle synchronization of responses to the dynamic FE videos across trials. Visual inspection showed two separate event-related responses, one after video onset (0 ms), and a second around 1000 ms into stimulus presentation. Statistical analyses focused on comparing data extracted from these two time windows (TW, see Methods for details) for both power and phase-locking analysis across conditions and stimulus categories.

#### Event-related power analysis

**Fig. 2:**
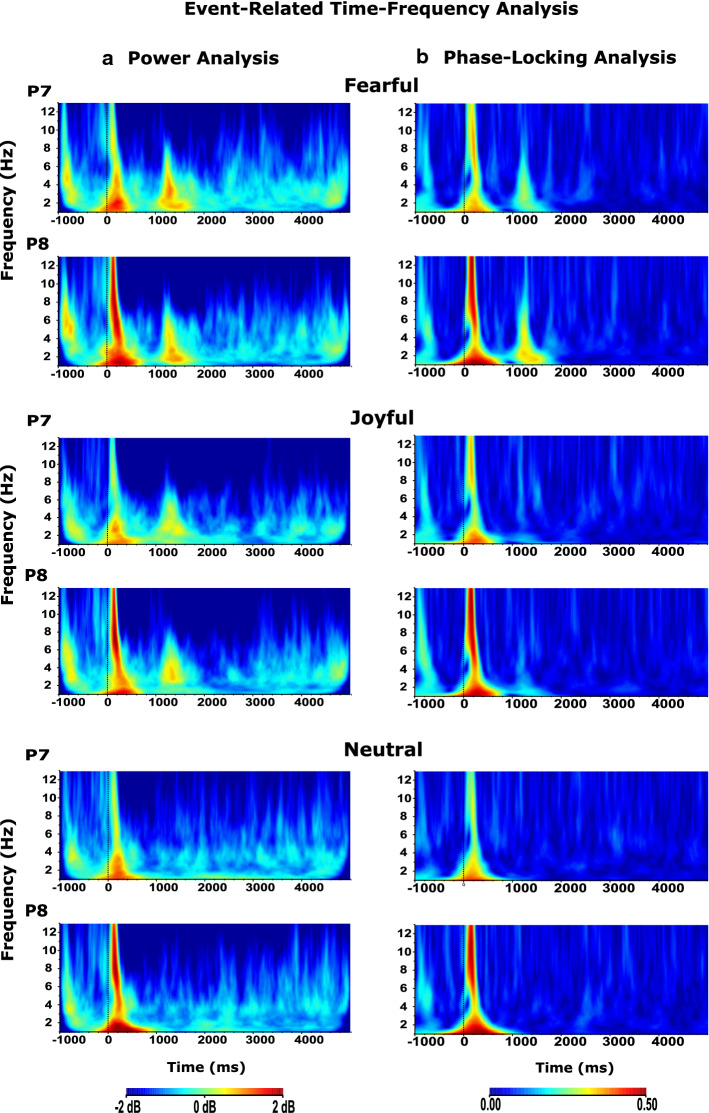
The grand average figures of event-related **a** power and **b** phase-locking analysis (1–13 Hz) in time–frequency domain in response to each dynamic FE categories which are given in figure, respectively: fearful, joyful, and neutral FEs. The parietal areas (p7–p8) were presented in the figure. **a** Delta and theta power in response to the emotional FEs (fearful and joyful FE) was higher than that of the neutral FE in the second time window that corresponds to the time between 1000 and 1800 ms for delta, 1000 ms and 1400 ms for theta (*p* < 0.05). In the first time window (0–400 ms), the right parietal-2 location (p8) had higher theta power (*p* < 0.05), while there was no hemispheric difference in the second time window (1000–1400 ms) (*p* > 0.05). In the time window between 0 and 250 ms that corresponds to the first time window, the right parietal-2 (p8) location had higher alpha power (*p* < 0.05), while there was no hemispheric difference in the second time window (1000–1250 ms) (*p* > 0.05). **b** Delta and theta phase-locking value in response to the fearful FE was higher than that of the joyful and the neutral FE in the second time window that corresponds to the time between 1000 and 1800 ms for delta, 1000 ms to 1400 ms for theta (*p* < 0.05). In the first time window (0–400 ms), the right parietal-2 location (p8) had a higher delta, theta, and alpha phase-locking value (*p* < 0.05), while there was no hemispheric difference in the second time window (*p* > 0.05). The X-axis represents time, and the Y-axis represents frequency; the point at which the stimulus arrives is marked as a zero point on the X-axis. p7–p8: parietal

A repeated-measures ANOVA on mean normalized power included factors Time (first TW, second TW), FE (neutral, joyful, fearful), Location (seven electrode clusters, see Methods), and Hemisphere (left, right). Figure 2a shows the event-related power changes in the time–frequency domain (1–13 Hz) as a response to our dynamic FEs per stimulus category for left and right parietal-2 electrodes (P7, P8) in which the main differences were observed. By eye, the two time windows with distinct responses can be observed around 0 and 1000 ms into the stimulus videos.

##### Delta frequency

There was a main effect of Time (*F*(df = 1, 17) = 41.965, *p* = 0.001, and *η*_*p*^2^ = 0.712): the first TW had higher delta power than the second TW (Fig. 2). In addition, FE had an effect (*F*(df = 2, 34) = 3.406, *p* = 0.047, and *η*_*p*^2^ = 0.167): delta power in response to the emotional FEs (joyful, fearful) was higher than that of the neutral FE. This difference was due to the responses generated in the second TW, largely, and indeed the Time*FE interaction was statistically significant (*F*(df = 2, 34) = 7.415, *p* = 0.003, and *η*_*p*^2^ = 0.304). Post-hoc results showed that the delta power for emotional FEs in the second TW was higher than for neutral FE (*p* < 0.05) (Fig. 2). There was also a statistically significant Time*Location interaction (*F*(df = 6, 102) = 7.304, *p* = 0.003, and *η*_*p*^2^ = 0.301). Post-hoc results showed that occipital areas had the lowest delta power compared to other areas in the second TW (*p* < 0.05), while there was no such location differentiation in the first TW (*p* > 0.05). Time*Hemisphere interaction was statistically significant (*F*(df = 1, 17) = 4.747, *p* = 0.04, and *η*_*p*^2^ = 0.225). While there was no statistical difference between the hemispheres in the first TW (*p* > 0.05), delta power was higher in the left hemisphere than in the right hemisphere for the second TW (*p* < 0.05).

##### Theta frequency

There was a main effect of Time (*F*(df = 1, 17) = 79.534, *p* = 0.001, and *η*_*p*^2^ = 0.824): the first TW had higher theta power than the second TW (*p* < 0.05) (Fig. 2). The location had an effect (*F*(df = 6, 102) = 5.986, *p* = 0.001, and *η*_*p*^2^ = 0.260): theta power was highest in the occipital location (*p* < 0.05). Time*FE interaction was statistically significant (*F*(df = 2, 34) = 5.019, *p* = 0.012, and *η*_*p*^2^ = 0.228). While the responses for different FEs in the first TW did not differentiate (*p* > 0.05), the theta power for emotional FEs in the second TW was higher compared to the neutral FE (*p* < 0.05) (Fig. 2). There was a statistically significant Time*Location interaction (*F*(df = 6, 102) = 4.107, *p* = 0.025, and *η*_*p*^2^ = 0.195). Post-hoc results showed that the occipital area had higher theta power compared to the anterior areas in the second TW (*p* < 0.05), while there was no such location differentiation in the first TW (*p* > 0.05). In addition, Time*Location*Hemisphere interaction was statistically significant (F(df = 6, 102) = 3.989, *p* = 0.014, and *η*_*p*^2^ = 0.190). In the first TW, the right parietal-2 location had higher theta power (*p* < 0.05), while there was no hemispheric difference between the locations in the second TW (*p* > 0.05).

##### Alpha frequency

There was a main effect of Time (*F*(df = 1, 17) = 77.993, *p* = 0.001, and *η*_*p*^2^ = 0.821): the first TW had higher alpha power than the second TW (*p* < 0.05). There was a statistically significant Time*Location interaction (*F*(df = 6, 102) = 5.999, *p* = 0.002, and *η*_*p*^2^ = 0.261). Post-hoc results showed that parieto-occipital areas (parietal-2, occipital) had higher alpha power compared to the other areas in the first TW (*p* < 0.05), while there was no such location differentiation in the second TW (*p* > 0.05). Time*Hemisphere interaction was statistically significant (*F*(df = 1, 17) = 5.294, *p* = 0.034, and *η*_*p*^2^ = 0.237). Accordingly, there was no hemispheric differentiation in the second TW (*p* > 0.05), while the right hemisphere had higher alpha power than the left in the first TW (*p* < 0.05). In addition, the Time*Location*Hemisphere interaction was statistically significant (*F*(df = 6, 102) = 4.953, *p* = 0.007, and *η*_*p*^2^ = 0.226). The right temporo-parietal areas (temporal, parietal-2) had higher alpha power than the left temporo-parietal areas in the first TW (*p* < 0.05), while there was no such differentiation in the second TW (*p* > 0.05) (Fig. 2).

#### Event-related phase-locking analysis

A repeated-measures ANOVA on phase-locking value included factors Time (first TW, second TW), FE (neutral, joyful, fearful), Location (seven electrode clusters, see Methods), and Hemisphere (left, right). Figure 2b shows the event-related phase-locking values in the time–frequency domain (1–13 Hz) as a response to our dynamic FEs per stimulus category for left and right parietal-2 electrodes (P7, P8) in which the main differences were observed. By eye, the two time windows with distinct responses can be observed around 0 and 1000 ms into the stimulus videos as in the power analysis.

##### Delta frequency

There was a main effect of Time (*F*(df = 1, 17) = 67.762, *p* = 0.001, and *η*_*p*^2^ = 0.799): the first TW had a higher delta phase-locking value than the second TW (Fig. 2). In addition, FE had an effect (F(df = 2, 34) = 14.701, *p* = 0.001, and *η*_*p*^2^ = 0.464): delta phase in response to the fearful FEs was higher than that of the joyful and neutral FE (*p* < 0.05). This difference was due to the responses generated in the second TW, largely, and accordingly, the Time*FE interaction was statistically significant (F(df = 2, 34) = 24.748, *p* = 0.001, and *η*_*p*^2^ = 0.593). Post-hoc results showed that the delta phase-locking value for fearful FEs in the second TW was higher than for joyful and neutral FE (*p* < 0.05), while there was no such FE differentiation in the first TW (*p* > 0.05) (Fig. 2). The location had an effect (*F*(df = 6, 102) = 11.841, *p* = 0.001, and *η*_*p*^2^ = 0.411): delta phase-locking value was highest in the parietal-2 and occipital locations (*p* < 0.05). This location difference is due to the delta responses in the first time window owing to the fact that there was a statistically significant Time*Location interaction (*F*(df = 6, 102) = 6.547, *p* = 0.004, and *η*_*p*^2^ = 0.278). Post-hoc results showed that parietal-2 and occipital location areas higher compared to other areas in the first TW (*p* < 0.05), while there was no such location differentiation in the second TW (*p* > 0.05). Hemisphere had an effect (*F*(df = 1, 17) = 18.273, *p* = 0.001, and *η*_*p*^2^ = 0.518): right delta phase-locking value was higher than in the left (*p* < 0.05). Time*Hemisphere interaction was statistically significant (*F*(df = 1, 17) = 4.602, *p* = 0.047, and *η*_*p*^2^ = 0.213). While there was no statistical difference between the hemispheres in the second TW (*p* > 0.05), the delta phase-locking value was higher in the right hemisphere than in the left hemisphere for the first TW (*p* < 0.05). Location*Hemisphere interaction was statistically significant (*F*(df = 6, 102) = 6.843, *p* = 0.001, and *η*_*p*^2^ = 0.287). Consequently, the right temporo-parietal areas (temporoparietal, parietal-2) had higher delta phase-locking values than in the left (*p* < 0.05). This difference was due to the responses generated in the first TW and Time*Location*Hemisphere interaction was statistically significant (*F*(df = 6, 102) = 3.888, *p* = 0.024, and *η*_*p*^2^ = 0.186). Post-hoc results showed that the delta phase-locking value in the right temporo-parietal areas was higher than in the left for in the first TW (*p* < 0.05), while there was no such differentiation in the second TW (*p* > 0.05) (Fig. 2).

##### Theta frequency

There was a main effect of Time (*F*(df = 1, 17) = 127.587, *p* = 0.001, and *η*_*p*^2^ = 0.882): the first TW had higher theta phase-locking value than the second TW (Fig. 2). FE had an effect (*F*(df = 2, 34) = 9.857 701, *p* = 0.002, and *η*_*p*^2^ = 0.367): theta phase in response to the fearful FEs was higher than that of the joyful and neutral FE (*p* < 0.05). This difference was due to the responses generated in the second TW, largely, and accordingly, the Time*FE interaction was statistically significant (*F*(df = 2, 34) = 9.166, *p* = 0.001, and *η*_*p*^2^ = 0.350). Post-hoc results showed that the theta phase-locking value for fearful FEs in the second TW was higher than for joyful and neutral FE (*p* < 0.05), while there was no such FE differentiation in the first TW (*p* > 0.05) (Fig. 2). The location had an effect (*F*(df = 6, 102) = 7.844, *p* = 0.001, and *η*_*p*^2^ = 0.316): theta phase-locking value was highest in the occipital locations (*p* < 0.05). This location difference is due to the theta responses in the first time window and consequently, there was a statistically significant Time*Location interaction (*F*(df = 6, 102) = 3.942, *p* = 0.012, and *η*_*p*^2^ = 0.188). Post-hoc results showed that occipital areas higher compared to other areas in the first TW (*p* < 0.05), while there was no such location differentiation in the second TW (*p* > 0.05). Hemisphere had an effect (*F*(df = 1, 17) = 4.466, *p* = 0.05, and *η*_*p*^2^ = 0.208): right theta phase-locking value was higher than in the left (*p* < 0.05). Time*Location*Hemisphere interaction was statistically significant (*F*(df = 6, 102) = 3.080, *p* = 0.035, and *η*_*p*^2^ = 0.153). Post-hoc results showed that the theta phase-locking value in the right temporo-parietal areas (temporoparietal, parietal-2) was higher than in the left for in the first TW (*p* < 0.05), while there was no such differentiation in the second TW (*p* > 0.05) (Fig. 2).

##### Alpha frequency

There was a main effect of Time (*F*(df = 1, 17) = 71.155, *p* = 0.001, and *η*_*p*^2^ = 0.807): the first TW had higher alpha phase-locking value than the second TW (Fig. 2). The location had an effect (*F*(df = 6, 102) = 11.823, *p* = 0.001, and *η*_*p*^2^ = 0.410): alpha phase-locking value was highest in the parietal-2 and occipital locations (*p* < 0.05). Hemisphere had an effect (*F*(df = 1, 17) = 6.967, *p* = 0.017, and *η*_*p*^2^ = 0.291): right alpha phase-locking value was higher than in the left (*p* < 0.05). In addition, Location*Hemisphere interaction was statistically significant (*F*(df = 6, 102) = 6.792, *p* = 0.002, and *η*_*p*^2^ = 0.285). Post-hoc results showed that the right temporo-parietal areas (temporoparietal, parietal-2) had higher alpha phase-locking values than in the left (*p* < 0.05). According to post-hoc comparisons, this location, hemisphere difference, and Location*Hemisphere interaction is due to the alpha responses, mainly, in the first time window (0 < 0.05), and consequently, there were statistically significant Time*Location interaction (*F*(df = 6, 102) = 8.314, *p* = 0.001, and *η*_*p*^2^ = 0.328), Time*Hemisphere interaction (*F*(df = 1, 17) = 10.574, *p* = 0.005, and *η*_*p*^2^ = 0.383), and Time*Location*Hemisphere interaction (*F*(df = 6, 102) = 4.774, *p* = 0.011, and *η*_*p*^2^ = 0.219). In addition, Time*FE*Hemisphere interaction was statistically significant (*F*(df = 2, 34) = 4.872, *p* = 0.018, and *η*_*p*^2^ = 0.223). Post-hoc results showed that the alpha phase-locking value in the right hemisphere for the neutral FE was higher than in the left in the first TW (*p* < 0.05), while there was no such differentiation in the second TW (*p* > 0.05).

**Fig. 3:**
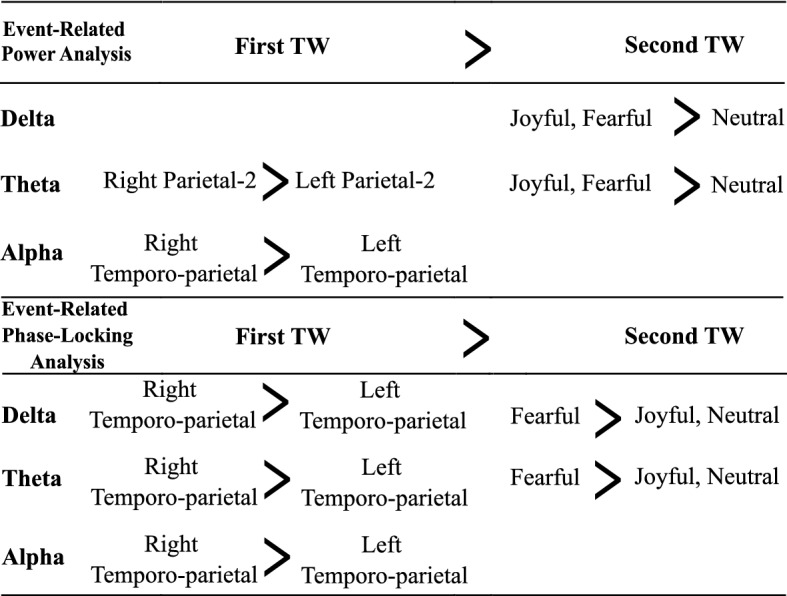
Summary of the main results. Temporo-parietal areas include the temporo-parietal and parietal-2 locations. Hem: hemisphere, TW: time window

A summary representation of the main results of the study is given in Fig. 3.

## Discussion

We here investigated the neuronal processing of dynamic faces progressing from neutral to fearful, joyful, or continued neutral expressions. Our main interest was to evaluate to what extent event-related oscillations might show a similar temporal progression, simultaneously revealing the feasibility of EEG oscillation studies using such ecologically valid, but more complex, dynamic stimuli. The cardinal findings of the present study were as follows: (I) an early time window (first TW) had higher power and phase-locking values than a later time window (second TW) for all frequency bands; (II) delta and theta power were higher in response to the emotional FEs than the neutral, in the second TW; (III) delta and theta phase-locking values were higher in response to the fearful FEs than to joyful and neutral FEs, in the second TW; and (IV) the right parietal locations had higher power and phase-locking value in the first TW, especially for the theta and alpha frequencies, while there was no such differentiation in the second TW. These results show that event-related oscillations are sensitive to the temporal aspect of FE processing as we hypothesized. In addition, differentiation in the response to emotional faces was shown in later time windows, as we expected.

Previous studies emphasized that facial processing in the brain involves a core temporal aspect, due to the dynamic nature of facial expressions [35–37]. In this study, we found that the neuronal response mainly occurred in two different time windows during dynamic FE processing, with a stronger response in the first TW for all frequency bands. Responses appearing in the second TW correspond approximately to the onset of emotional expressions in the video clips from the joyful/fearful categories. Therefore, that specifically in the emotional FE stimuli, the stimulus noticeable changed, while in the neutral video, it did not. As summarized in Fig. 3, however, oscillation responses in the second TW were different specifically for the fearful videos, arguing against the idea that the second TW responses might be simply due to low-level feature changes in the visual input. Overall, these results imply that there could be two main stages to FE processing, which could reflect initial sensory/perception, and then recognition/judgment. The fact that these responses were segregated in time underlines the added value of using dynamic FE stimuli, where the onset of a face stimulus and the onset of an emotional expression can be temporally separated.

That the sensory response (first TW) was higher in power and phase-locking than the higher order response (second TW) may be related to the fact that the brain will always respond more synchronized to the sudden onset of a stimulus, while the more subtle and gradual change into an emotional expression has a less clear/sudden temporal onset which means no phase reset and more differences across participants.

In the present study, participants identified the highest arousal and valence score for the joyful FE and performed better in the post-hoc classification of these stimuli as joyful. Results of the current study showed that the emotional FEs (joyful and fearful) had higher delta and theta power compared to the neutral, in the second TW. In addition, results showed that responses generated for the fearful FE were more phase-locked than the joyful and neutral FE. The differentiation with respect to emotion was observed in the later time window which might correspond to the higher order face processing stages, as hypothesized. This differentiation was represented by delta and theta oscillations, which is in accordance with the literature: the role of temporal–parietal–occipital delta responses in FE recognition has already been identified [19]. It is also known that the delta oscillation, especially the posterior delta, may have an important role in the perception and recognition of complex images and also may reflect episodic memory processes [22]. Yet, the results of the current study imply that delta oscillations are indeed sensitive to the emotional content of the stimuli, since all stimulus categories here could be considered ‘complex’.

The perceived emotional intensity of FEs is known to be influential in emotion processing in the brain [18, 26, 38–41]. Studies showed that FEs with higher arousal levels elicit higher neuronal activity [38–41]. In event-related oscillation studies, valence has been found to be represented mostly by higher frequencies and arousal to be represented by lower ones such as delta and theta [19]. Klados et al. [23] and Balconi et al. [27, 28] reported that delta responses were stronger to highly arousing pictures than to less arousing ones. Moreover, several studies showed that high-arousal stimuli result in stronger theta synchronization than low-arousal stimuli, which suggests that arousal discrimination could be represented by an increase in theta power [20, 24, 29, 42]. However, contrary to previous studies, the brain responses to the most arousing FE (joyful), did not differ significantly from the response to the fearful FE. The power of the brain responses to FEs was in line with their subjective arousal levels, even if it is statistically non-significant for the joyful–fearful comparison. In addition, a different pattern than expected was revealed for the phase-locked responses considering this arousal hypothesis, since the brain responses in response to fearful FEs were differentiated from the joyful and neutral FEs in terms of phase-locking values. It is also reported that there are specific neuronal localizations for the subjective feelings of valence and arousal rather than discrete topologies for discrete emotions [43, 44]. Accordingly, delta, theta, and alpha may topographically differentiate, rather than in terms of decrements/increments in the event-related responses according to the perceived valence and arousal of the fearful FE and the joyful FE. In order for this differentiation to be better emphasized, a larger sample population recruitment might be necessary.

Another main finding concerns lateralization which differs with respect to time and location. The posterior regions of the right hemisphere responses were higher in the first TW, especially at the parietal areas. All three frequencies had higher power and phase-locking values at the right parietal location in the first TW. Lateralization is established in the literature on face processing. It has been shown that there is a right-dominant neuronal mechanism in FE processing. Furthermore, several studies related FE perception to widespread neuronal processing in the right hemisphere, with an organization similar to language processing in the left hemisphere [45–48]. Aside from pioneering functional magnetic resonance imaging (fMRI) studies on FE processing, there are also EEG studies demonstrating this hemispheric lateralization during FE processing [8, 49–52]. However, such results were obtained with stimulus sets that mostly used static photographs, where the temporal dynamics of face processing were not considered [45, 46, 53]. According to the valence hypothesis [49, 50, 54], increased left anterior activity is associated mostly with positive emotions, while increased right anterior activity is associated with negative emotions. However, in this study, we see that the hemispheric difference is in posterior regions rather than anterior regions, and this hemispheric difference is not between the emotions (joyful vs. fearful), which is in line with Güntekin et al. [8]. Therefore, rather than the valence hypothesis, the results support the right hemisphere hypothesis [51, 55], which suggests that the right hemisphere is important in processing emotions irrespective of their valence. The present study also showed that the lateralization differs over time during dynamic FE processing, which may reflect the different stages inherent to FE processing as discussed above. Such stages may include visual perception, the formation of high-level representations of the face, and the recognition of the emotion or the identity [43], all of which are hierarchically spread both in time and topology. It is well-known that different oscillations reflect different components of such sensory and emotional processes; for reviews see Knyazev’s [17] and Güntekin & Başar [18].

This study has several limitations that should be considered for future studies. The sample size was relatively small, and electromyographic (EMG) activity was not recorded although it might provide valuable information. Previous studies showed that facial muscle activity relates to comprehension of emotional state [56–59]. Furthermore, in this study, dynamic FE responses were not compared with static FE responses directly. Instead, the results of this study were discussed in comparison to the results of previous studies with static FEs from our group and others. Finally, future research could investigate the temporal aspects of dynamic FE processing within a social context.

## Conclusions

The present study revealed electroneurophysiology correlates of the processing of the dynamic facial expressions. Apparently, there are temporal changes during FE processing that will be missed in studies in which static FE stimuli are used. The current study showed that neuronal activity in response to facial expressions changes over time, and this is mainly observed in two distinct time windows. The neuronal response in the later time window was interpreted to indicate higher level processing, where the response to emotional faces vs. neutral faces is differentiated by activity in the delta–theta frequency range. In addition, in accordance with previous literature, we observed right parietal dominance during facial processing for all three frequency bands. In conclusion, facial expression processing has a temporal aspect that should be considered in studies that research FE processing.

## Methods

### Participants

A total of 18 healthy young participants (12 females, age: 22.2 ± 2, education (year): 16 ± 1.9) were included in the study. All the participants had a normal or corrected-to-normal vision and were right-handed. Having been diagnosed with or having a history of any neurological or psychiatric disease, using any neurological or psychiatric medicine were exclusion criteria. We obtained written informed consent from all participants. The ethics committee of Istanbul Medipol University approved the study (no: E47609).

### Experimental design and procedure

**Fig. 4:**
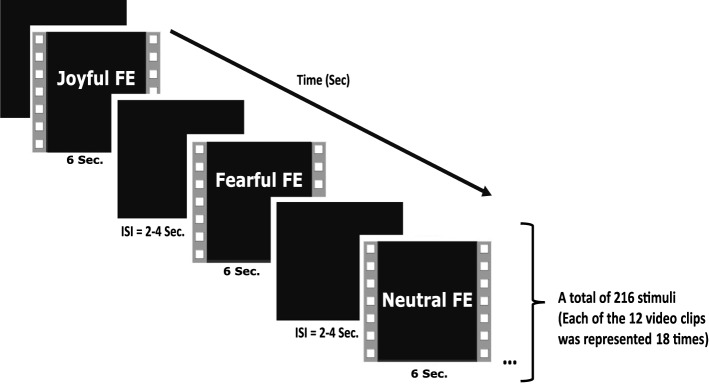
Representation of the experimental design. FE: facial Expression, ISI: inter-stimulus interval, Sec.: second

12 videos showing joyful, fearful, and neutral facial expressions were chosen from the Amsterdam Dynamic Facial Expression Set (ADFES) as stimuli [60]. Each of the chosen facial expressions was displayed by two different male and female models. Thus, the dynamic FE paradigm displayed during the EEG recording consists of 12 videos showing 3 facial expressions (joyful, fearful, neutral). Each of the 12 different videos was presented 18 times in the paradigm (a total of 216 stimuli were presented, randomly) (Fig. 4). The videos have a 6 s standard length. All the videos start with the neutral FE maintained for 0.5 s, and then the FE appears and is maintained for approximately 5 s [60]. Inter-stimulus intervals varied from 2 to 4 s, randomly. E-prime software (Psychology Software Tools Inc., Pittsburgh, PA) was used in the preparation and presentation of the task. The videos were presented in full screen on a monitor with a 60 Hz refreshing rate (47.5 × 26.8 cm) placed 90 cm away from participants.

After the EEG recording session, participants were asked to classify the FE expressed in each of the 12 videos as either neutral, fearful, or joyful. One point was given for each correct answer, which means that, with unique 4 video stimuli per category, there could be a maximum ‘FE recognition’ score of 4 (minimum of 0) for each category (neutral, joyful, fearful), and 12 points in total across the three categories. The subjective valence and arousal level of subjects in response to the stimuli presented in each FE may vary. Although it has been shown that the EEG event-related responses may differ according to the category of FE stimuli, this differentiation may be due to the subjective valence and arousal of individuals rather than any categorical distinction of FEs. Since there are several studies showing that the neuronal processing of the stimuli could be differentiated by subjective arousal and valence recognition [18, 38–42], in this study participants were asked to rate the valence and arousal for each of the 12 video stimuli in the experiment, using SAM (Self Assessment Manikin) images [61]. For arousal level evaluation, the nine-point rating SAM scale was used, where one point showed the lowest excitation state, and nine points showed the highest excitation state. For valence level evaluation, the 9-point rating SAM scale was used, where 1–4 points represent negative [1], 5 points represent neutral, 6–9 points represent positive [9]. These applied metrics gave us the opportunity to test whether the differentiation in EEG responses is in line with the subjective valence and arousal scores.

### EEG recording procedure

EEG recording was amplified with a BrainAmp 32-Channel DC System (Brain Product GmbH, Germany). Fp1, Fp2, F7 F3, Fz, F4, F8, Ft7, Fc3, Fcz, Fc4, Ft8, Cz, C3, C4, T7, T8, Tp7, Cp3, Cpz, Cp4, Tp8, P3, Pz, P4, P7, P8, O1, Oz, and O2 electrodes were recorded. The sampling rate of the EEG recording was 500 Hz and recording was performed with band limits of 0.01–250 Hz. EEG recording was performed using the “BrainCap with Multitrodes” model cap (EasyCap GmbH, Germany), which was arranged according to the international 10/20 system and has 32 electrode locations. In addition, two connected electrodes (A1 + A2) were placed on the earlobes as a reference. Electrooculogram (EOG) was recorded through electrodes placed on the medial upper and lateral orbital area of the left eye to identify eye movement in the EEG data. All electrode impedance values ​​were kept below 10 kOhm. Participants were sat in a dimly lit, soundproof, electrically shielded room during the entire experiment.

### EEG data analysis

#### EEG pre-processing steps

Data were preprocessed in the Brain Vision Analyzer (BVA) 2.2 software, before the advanced analysis. Preprocessing steps were applied, respectively, as follows: (I) data were digitally filtered by the eigth-order zero phase shift Butterworth filters (the infinite impulse response filters) between 0.01 and 60 Hz. (II) Independent component analysis was applied to the whole data to subtract eye movement components. Maximally two components were eliminated for the horizontal and vertical eye movements considering their topology, voltage ranges, and the respective improvement in the data following the extraction of these components. (III) Data were segmented considering stimulus onset. Since the FE was held for 5 s in the stimuli, data were divided into segments as 1 s before the stimulus and 5 s after the stimulus. The total duration of the segments was 6 s. (IV) Artifact rejection was applied to the segmented data. All data were manually checked, epoch by epoch. Epochs with artifacts (e.g., muscle activity) were removed.

EEG analyses were performed for the joyful, fearful, and neutral FEs separately. Studies with facial expression paradigms have shown the importance of posterior areas in facial expression processing, as mentioned in the Introduction; therefore, different posterior electrode pairs were chosen for the analysis. Yet, to see topographical changes during facial expression processing, some other important locations were added too. Ultimately, seven locations, namely 14 electrodes [frontal (F3–F4), central (C3–C4), temporal (T7–T8), temporoparietal (Tp7–Tp8), parietal-1 (P3–P4), parietal-2 (P7–P8), occipital (O1–O2)] were chosen for further EEG analysis, since these locations covered all cortical areas of interest.

#### Event-related time–frequency analyses

We applied event-related phase-locking and power analyses to the EEG data in time–frequency analysis. BrainVision Analyzer 2.2 software was used for the wavelet transform (WT). For each subject, each FE, and each electrode, continuous WT with Gabor normalized complex Morlet wavelet was applied over the preprocessed data. Single trials that WT was applied to were averaged for evaluation of the event-related total power and event-related phase-locking analysis.

Wavelet and output normalization parameters for the event-related power analysis are given in Table 1. This analysis shows the total power (both evoked and induced power) for a certain frequency at a certain time for the event-related responses both phase-locked and non-phase-locked [61]. The same wavelet parameters were used for the event-related phase-locking analysis as can be seen in Table 1. Phase-locking analysis assesses the consistency of the phase angle of signals across trials. Results of the phase-locking analysis do not include the power (amplitude/magnitude) features of the signal. Phase-locking values vary between 0 and 1. Values close to zero represent the random phase angles between the trials, while phase-locking values close to one refers to perfect phase alignment across the trials.

**Table 1:** Wavelet parameters for power and phase-locking analysis

	Delta	Theta	Alpha
Frequency Band	1–3.5 Hz	4–7 Hz	8–13 Hz
Frequency step (logarithmic step)	60	60	60
Morlet parameter c (cycle)	3	3	3
Wavelet output normalization^a^			
Output values	Spectral power (μV^2^)	Spectral power (μV^2^)	Spectral power (μV^2^)
Normalization method	Decibel (dB)	Decibel (dB)	Decibel (dB)
Reference Interval (ms)	start -500, end -300	start -500, end -300	start -500, end -300

^a^Output normalization methods were applied only for power analysis

Numerical values of the calculated WT were exported. Exports were done through two different time windows for each frequency band, namely: 0–800 ms and 1000–1800 ms for delta (1–3.5 Hz), 0–400 ms, and 1000–1400 ms for theta (4–7 Hz) and 0–250 ms and 1000–1250 ms for alpha (8–13 Hz) frequency bands. The mean power and phase values in these specific time windows were used in statistical analyses.

### Statistical analysis

SPSS 22 software was used for statistical analyses. Repeated Measures ANOVAs were performed on the mean values of normalized event-related power and the mean values of event-related phase-locking in the two time windows, separately per frequency band (delta, theta, and alpha). ANOVAs were employed with four within-group factors; time window (first TW, second TW), FE (joyful, fearful, and neutral), location (frontal, central, temporal, temporoparietal, parietal-1, parietal-2, and occipital), and hemisphere (left and right). Greenhouse–Geisser corrected *p* values were reported. Post-hoc analyses were accomplished with Statistica software. Bonferroni correction for multiple comparisons was used for the post-hoc tests. Kruskal–Wallis test was used to test the significance of the difference between the FEs’ valence, arousal, and recognition scores. The significance level was determined as *p* < 0.05.

## Data Availability

The datasets used and/or analyzed during the current study are available from the corresponding author on reasonable request.
